# Incidence and Prognostic Value of TP53, STK11, and KEAP1 Mutations Between *De Novo* Versus Recurrent Actionable Mutation–Negative Non-Small Cell Lung Cancer: A Single-Center Retrospective Study

**DOI:** 10.14740/wjon2761

**Published:** 2026-05-08

**Authors:** Jorge Raul Vazquez-Urrutia, Natasha Venugopal, Junjia Zhu, Takefumi Komiya

**Affiliations:** a Department of Medicine, Penn State Health Milton S. Hershey Medical Center, Hershey, PA, USA; b Division of Hematology Oncology, Penn State College of Medicine, Hershey, PA, USA; c Department of Public Health Sciences, Penn State College of Medicine, Hershey, PA, USA

**Keywords:** NSCLC, NGS, Actionable mutations, TP53, STK11, KEAP1

## Abstract

**Background:**

In non-small cell lung cancer (NSCLC), mutations in TP53, STK11, and KEAP1 are common in tumors lacking actionable oncogenic drivers and have been associated with poor outcomes, though their prognostic impact remains context-dependent. We evaluated the incidence and prognostic significance of these mutations in actionable mutation–negative NSCLC, stratified by *de novo* versus recurrent disease.

**Methods:**

We retrospectively analyzed 119 adult patients with NSCLC and available next-generation sequencing (NGS) results treated at our center through 2024. Cases with actionable mutations were excluded. Patients were classified as *de novo* (n = 82) or recurrent (n = 37) based on the disease status at the point of analysis. Between-group comparisons were done using Fisher’s exact and Wilcoxon Rank-sum test. Overall survival and progression-free survival were assessed. All tests were two-sided and a P value < 0.05 was considered statistically significant.

**Results:**

TP53 mutations were most frequent (61% *de novo* vs. 54% recurrent; P > 0.05). STK11 and KEAP1 mutations occurred at similar rates between groups (5–15%; P > 0.05). Baseline clinical characteristics were balanced. No significant differences in overall or progression-free survival were observed by mutational status in either cohort.

**Conclusion:**

In this real-world cohort of actionable mutation–negative NSCLC cases, we did not detect significant prognostic associations for TP53, STK11, and KEAP1 mutations, and their incidence was similar between *de novo* and recurrent disease. These findings underscore the need for larger studies to evaluate the prognostic utility of these mutations in this clinical context.

## Introduction

Lung cancer remains the leading cause of cancer-related mortality worldwide, with non-small cell lung cancer (NSCLC) accounting for approximately 85% of all cases [1]. Advances in genomic profiling have significantly transformed the management of NSCLC over the past decade. Next-generation sequencing (NGS) is a comprehensive mode of molecular analysis that provides detailed genomic profiling of patients’ malignancies [2]. In the context of NSCLC, NGS has deepened the understanding of its heterogeneous landscape, allowing for the discovery of targetable mutations such as EGFR, ALK, and ROS1. The development of oncogene-driven therapies has significantly improved overall survival (OS) and progression-free survival (PFS) in selected patients [2–4]. For example, in patients with EGFR-mutant NSCLC treated with first-line osimertinib, the 3-year overall survival rate has been reported to be approximately 54% [4]. Furthermore, in a study of 200 patients, those treated with targeted therapy had a longer median OS (mOS) and PFS of 26.2 and 13.4 months, respectively, compared to patients who received chemotherapy, who had an mOS and mPFS of 8.8 and 5.2 months, respectively [2].

Despite these advancements, a substantial proportion of patients with NSCLC lack known actionable mutations. In a cohort of 200 patients, 35% were found to have no known actionable mutations by NGS [2]. Similarly, a study of 1,007 patients with lung adenocarcinoma demonstrated that only 64% of patients harbored actionable mutations [5]. For patients without identifiable mutations, treatment typically relies on immunotherapy with or without chemotherapy. However, outcomes with these strategies remain inferior compared to those observed in patients receiving oncogene-driven treatment [6]. Long-term response rates with first-line immunotherapy alone and with chemoimmunotherapy remain limited [7]. For example, the 5-year OS was only 21.9% among NSCLC patients with programmed death ligand-1 Tumor Proportion Score (PD-L1 TPS) ≥ 50% who received 35 cycles of pembrolizumab [8]. In another study of 616 patients, the 5-year OS rate was 19.4% for patients receiving chemo-immunotherapy and 11.3% for patients receiving chemotherapy alone [9].

Many tumors without actionable driver mutations instead demonstrate alterations in tumor suppressor genes such as TP53, STK11, and KEAP1. Current evidence suggests that mutations in these genes may be negative prognostic factors in NSCLC. For instance, TP53 mutations have been independently associated with worse OS in metastatic disease, while KEAP1 mutations are associated with worse outcomes in surgically resected disease [10]. Similarly, STK11-mutant tumors—particularly those with cooccurring TP53 and KRAS mutations—show more lymphovascular invasion and concurrent immunotherapy resistance [7]. However, the clinical significance of these mutations remains uncertain and appears to be context-dependent, with variations in prognostic role influenced by factors such as stage, histology, and actionable mutation status [6, 8]. Furthermore, these mutations have been studied mostly in advanced disease, and their significance in predicting recurrence after curative-intent therapy in actionable mutation–negative cases remains understudied.

In this study, we aimed to analyze the incidence and prognostic significance of TP53, STK11, and KEAP1 mutations in patients with NSCLC who lack actionable mutations, stratified by *de novo* versus recurrent disease presentation, to determine whether these are enriched in recurrent disease and may serve as risk stratifiers that would guide treatment decisions in this unique population.

## Materials and Methods

This is a retrospective, observational study including NSCLC patients diagnosed and treated at our center between 2017 and 2024. Patients who underwent tissue biopsy and molecular profiling using the commercially available CARIS molecular platform—which provides comprehensive molecular profiling that covers DNA (NGS-based whole exome sequencing), RNA (whole transcriptome sequencing), and proteins (immunohistochemistry)—for thoracic malignancies and were treated at our center, were manually screened. Additional clinical and pathologic data were extracted from the electronic medical record. This study was reviewed by the Penn State Health Institutional Review Board (IRB #00026156) and was designated exempt from human subjects research.

Eligible cases consisted of adult patients who were diagnosed and treated at our institution and had available molecular profiling data. Exclusion criteria included age < 18 years, pregnancy, incarceration, and the presence of actionable driver alterations, including EGFR, ALK, ROS1, NTRK, KRAS, BRAF, RET, MET, or HER2 mutations.

After applying inclusion and exclusion criteria, patients were stratified by disease status into two groups: *de novo* disease and recurrent disease. *De novo* disease was defined as NSCLC diagnosed at any stage at initial presentation, without prior definitive treatment. Recurrent disease was defined as radiographic or clinical re-emergence of disease following a documented complete response after standard-of-care therapy.

Among patients without actionable mutations, TP53, STK11, and KEAP1 were selected for further sub-stratification to assess mutation-specific clinical outcomes as their overall frequencies exceeded 5% in both groups and they are among the most commonly reported in the literature [11] (Supplementary Material 1, wjon.elmerpub.com).

The primary outcomes of interest were OS, defined as the time from initial diagnosis to death from any cause or last follow-up, and PFS, defined as the time from diagnosis to the first occurrence of disease progression or death from any cause.

Key clinical and demographic variables were collected and analyzed within each stratum. These included age, sex (male vs. female), race (White vs. other), smoking status (never vs. former vs. current), Eastern Cooperative Oncology Group (ECOG) performance status (0–1 vs. > 1), family history of lung cancer (yes vs. no), and comorbid conditions (heart failure, coronary artery disease, hypertension, chronic obstructive pulmonary disease, asthma, chronic kidney disease, diabetes, and other malignancies). Tumor-related variables included histologic subtype (adenocarcinoma vs. squamous vs. other histology), disease stage at diagnosis and recurrence (I–IV), treatment history, and PD-L1 (22c3) expression (0% vs. 1–49% vs. 50–100%).

### Statistical methods

Patients were stratified by disease state, and differences between groups were examined using Fisher’s exact tests or nonparametric Wilcoxon rank-sum tests when appropriate. Univariate and multivariable Cox proportional hazard regression models were used to calculate hazard ratios (HRs) and their 95% confidence intervals (CIs) for PFS and OS and independent prognostic factors were identified. Kaplan–Meier survival curves were generated and compared between disease and mutation strata using log-rank tests. All analyses were performed using JMP^®^ 14.0 (SAS Institute Inc., Cary, NC, USA) and R Programming Language version 4.5.2 (R Foundation for Statistical Computing, Vienna, Austria) [12]. All tests were two-sided and P < 0.05 was considered statistically significant.

## Results

**Figure 1:**
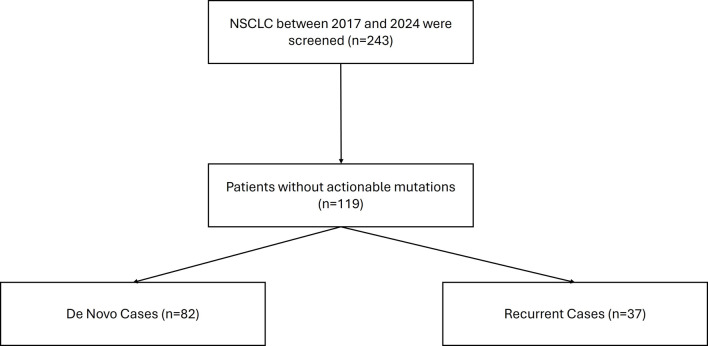
Consort diagram for case selection.

Our sample selection process is detailed in Figure 1. A total of 243 NSCLC patients were screened. After excluding 124 patients with actionable mutations, 82 and 37 patients were assigned to the *de novo* and recurrent groups, respectively. The clinical characteristics of the patients are shown in Table 1. Mean age at diagnosis was similar between the *de novo* and recurrent groups (68.1 vs. 68.6 years; P > 0.05). No significant differences were observed between groups with respect to age, smoking status, sex, race, ECOG performance status, comorbidities, family history, tumor histology, or PD-L1 expression. Stage at diagnosis was more advanced in the *de novo* group than in the recurrent group (stage IV: 63% vs. 2%; P < 0.001). Patients in the recurrent group were more likely to undergo surgery (57% vs. 8%; P < 0.001) and receive chemotherapy (86% vs. 66%; P = 0.02), while no significant differences were observed in the use of immunotherapy or radiation therapy (Table 1).

**Table 1:** Clinical Characteristics of Study Population

Factors	*De novo* (n = 82)	Recurrent (n = 37)	P value
Age at diagnosis			
Mean	68.1	68.6	0.67
Smoking status			
Never	11 (13%)	3 (8%)	0.7
Former	50 (61%)	25 (67%)	
Current	21 (26%)	9 (25%)	
Sex			
Male	48 (58%)	26 (70%)	0.31
Female	34 (42%)	11 (30%)	
Race			
White NH	74 (90%)	32 (87%)	0.54
Other	8 (10%)	5 (13%)	
ECOG at diagnosis			
0	26 (32%)	15 (41%)	0.63
1	34 (41%)	12 (32%)	
2	10 (12%)	6 (16%)	
3	7 (9%)	1 (3%)	
4	1 (1%)	0	
Unknown	4 (5%)	3 (8%)	
Number of comorbidities			
Mean	2.3	2.1	0.47
Family history of lung cancer			
Yes	17 (21%)	13 (35%)	0.11
No	65 (79%)	24 (65%)	
Histology			
Adeno	47 (57%)	21 (57%)	0.48
Squamous	22 (27%)	13 (35%)	
Others/unknown	16 (16%)	3 (8%)	
Stage on diagnosis			
I	3 (4%)	17 (46%)	< 0.001
II	5 (6%)	11 (30%)	
III	22 (27%)	8 (22%)	
IV	52 (63%)	1 (2%)	
Surgery			
Yes	7 (8%)	21 (57%)	< 0.001
No	75 (92%)	16 (43%)	
Radiation			
Yes	55(67%)	27 (73%)	0.66
No	27 (33%)	10 (27%)	
Chemotherapy			
Yes	54 (66%)	32 (86%)	0.02
No	28 (34%)	5 (13.5%)	
Immunotherapy			
Yes	53 (65%)	26 (70%)	0.68
No	29 (35%)	11 (30%)	
PD-L1 (22c3) expression			
Absent/0%	42 (51%)	17 (46%)	0.73
1–49%	23 (28%)	13 (35%)	
50–100%	17 (21%)	7 (19%)	
TP53 status			
Mutated	50 (61%)	20 (54%)	0.54
Wild-type	32 (39%)	17 (46%)	
STK11 status			
Mutated	8 (10%)	2 (5%)	0.72
Wild-type	74 (90%)	35 (95%)	
KEAP1 status			
Mutated	9 (11%)	6 (16%)	0.55
Wild-type	73 (89%)	31 (84%)	
KEAP1–STK11 co-mutation			
Yes	3 (4%)	2 (5%)	0.66
No	79 (96%)	35 (95%)	
TP53–STK11 co-mutation			0.31
Yes	4 (5%)	0	
No	78 (95%)	37 (100%)	
TP53–KEAP1 co-mutation			
Yes	5 (6%)	4 (11%)	0.66
No	77 (94%)	33 (89%)	

### Mutation frequency is similar in patients with *de novo* vs. recurrence NSCLC

TP53 was the most frequently detected alteration in both the *de novo* and recurrent groups (61% vs. 54%), although this difference was not statistically significant (P > 0.05). Similarly, the frequencies of STK11 (*de novo*: 10% vs. recurrent: 5%; P > 0.05) and KEAP1 (*de novo*: 11% vs. recurrent: 16%; P > 0.05) mutations did not differ significantly between groups (Table 1).

When stratified by disease presentation, KEAP1 and STK11 mutations demonstrated significant co-occurrence in both the *de novo* and recurrent groups (Table 2). In the *de novo* group, STK11 mutations were present in 33% of KEAP1-mutant tumors compared with 7% of KEAP1-wildtype tumors (Fisher’s exact P = 0.03; OR 6.53; 95% CI 0.82–45.01). In the recurrent cohort, STK11 mutations occurred in 33% of KEAP1-mutant tumors compared with 0% of KEAP1-wildtype tumors (Fisher’s exact P = 0.02); the odds ratio was not estimable due to a zero cell.

**Table 2:** STK11 and KEAP1 Co-Mutation Frequencies

Group	*De novo* (n = 82)				Recurrent (n = 37)			
	STK11 Neg	STK11 Pos	P value	OR	STK11 Neg	STK11 Pos	P value	OR
KEAP1 Neg	68 (93%)	5 (7%)	0.03	6.53 (0.82–45.01)	31 (100%)	0	0.02	NA
KEAP1 Pos	6 (67%)	3 (33%)			4 (67%)	2 (33%)		

Neg: negative; OR: odds ratio; Pos: positive.

In addition, TP53 mutation was not observed to be associated with STK11 or KEAP1 mutations (Table 3). The frequency of co-mutations did not differ significantly between *de novo* and recurrent cohorts (Table 1). Two cases harboring concurrent TP53, STK11, and KEAP1 mutations were identified in the *de novo* group, whereas none were observed among patients with recurrent disease.

**Table 3:** TP53 and STK11–KEAP1 Co-Mutation Frequencies

Group								
	STK11 Neg	STK11 Pos	P value	OR	KEAP1 Neg	KEAP Pos	P value	OR
*De novo* (n = 82)								
TP53 Neg	35 (90%)	4 (10%)	1	0.89 (0.22–3.58)	34 (89%)	4 (11%)	1	1.07 (0.28–4.02)
TP53 Pos	39 (91%)	4 (9%)			39 (89%)	5 (11%)		
Recurrent (n = 37)								
TP53 Neg	19 (90%)	2 (10%)	0.5	0.24 (0.01–5.28)	15 (88%)	2 (12%)	0.66	1.7 (0.31–9.2)
TP53 Pos	16 (100%)	0			16 (80%)	4 (20%)		

Neg: negative; OR: odds ratio; Pos: positive.

### Implications of TP53, STK11, and KEAP1 mutations on survival and progression

**Figure 2:**
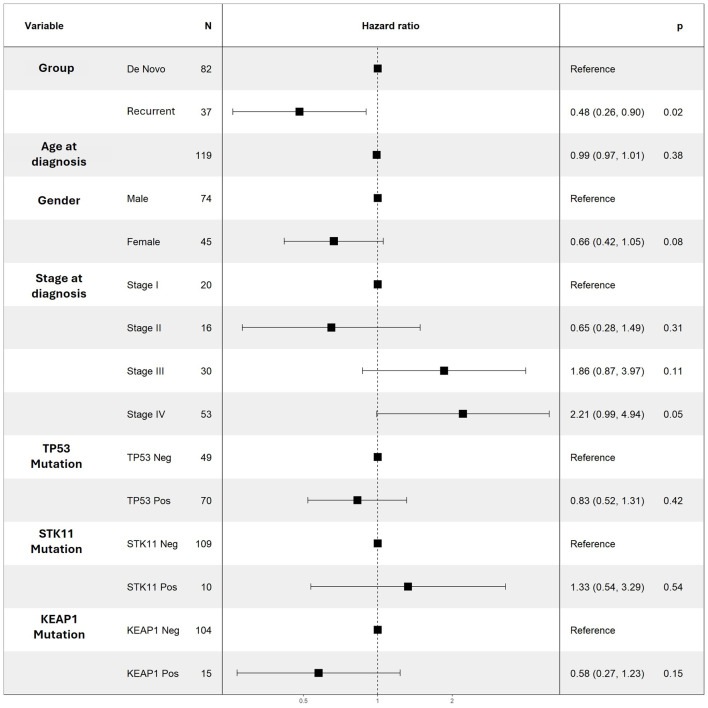
Multivariable Cox regression results for PFS for selected variables. No variable was noted to be a significant predictor of PFS. PFS: progression-free survival.

**Figure 3:**
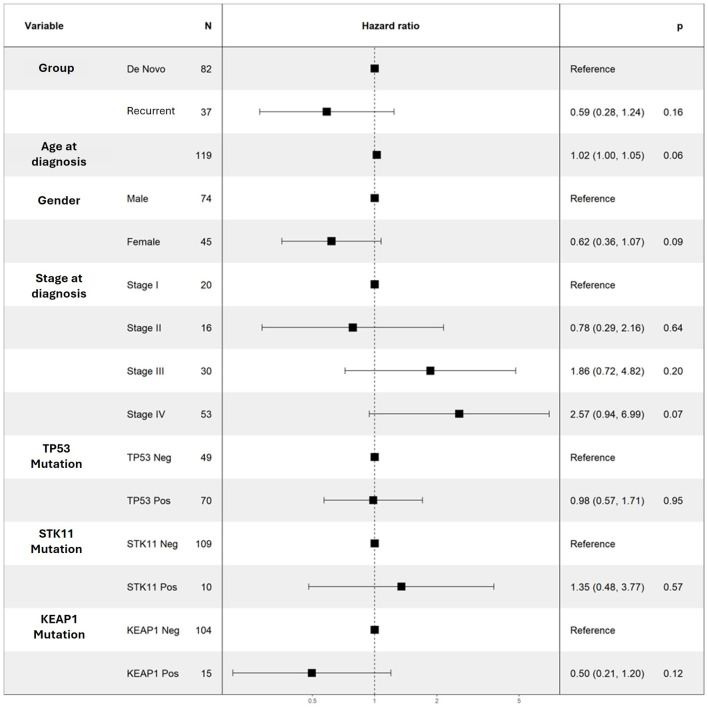
Multivariable Cox regression results for OS for selected variables. No variable was noted to be a significant predictor of OS. OS: overall survival.

When analyzing our entire cohort, neither univariate nor multivariate models demonstrated significant associations between TP53, STK11, or KEAP1 with PFS and OS (Table 2; Figs. 2 and 3). In addition, higher number of comorbidities was associated with lower hazard ratio of OS (HR 1.27; CI 1.09–1.47; P = 0.001) and PFS (HR 1.14; CI 1.001–1.29; P = 0.04) in all patients. Other demographic variables did not show consistent significant associations in the analyses (Table 4; Figs. 2 and 3).

**Table 4:** Univariate Cox Regression Results for Selected Variables

Factors	OS (HR)	PFS (HR)
Age at diagnosis	1.01 (1–1.03) P = 0.22	0.989 (0.97–1.009) P = 0.28
Number of comorbidities	1.27 (1.09–1.47) P = 0.001	1.14 (1.001–1.29) P = 0.04
Sex (female vs. male)	0.73 (0.44–1.19) P = 0.2	0.84 (0.55–1.26) P = 0.39
Race (non-White vs. White)	1.14 (0.55–2.4) P = 0.72	1.04 (0.55–2) P = 0.90
TP53 status (mutated vs. WT)	1.21 (0.75–1.95) P = 0.44	1.09 (0.73–1.63) P = 0.66
STK11 status (mutated vs. WT)	1.05 (0.42–2.6) P = 0.92	0.94 (0.43–2.02) P = 0.87
KEAP1 status (mutated vs. WT)	0.57 (0.26–1.24) P = 0.16	0.59 (0.30–1.13) P = 0.11
KEAP1–STK1 co-mutation (yes vs. no)	0.55 (0.13–2.26) P = 0.41	0.33 (0.08–1.34) P = 0.12

HR: hazard ratio; OS: overall survival; PFS: progression-free survival; WT: wild-type.

**Figure 4:**
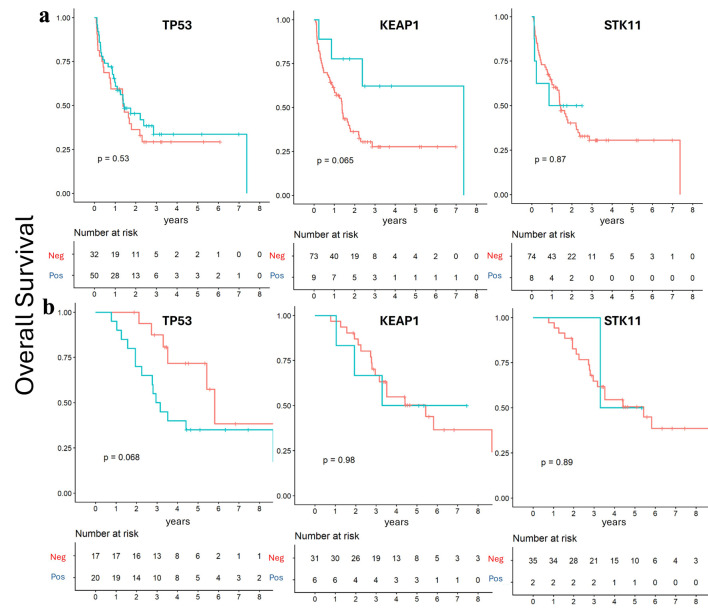
Overall survival according to mutation status. Kaplan–Meier survival analysis showed no significant survival differences according to TP53, STK11, and KEAP1 mutation status. (a) *De novo* group. (b) Recurrent group.

**Figure 5:**
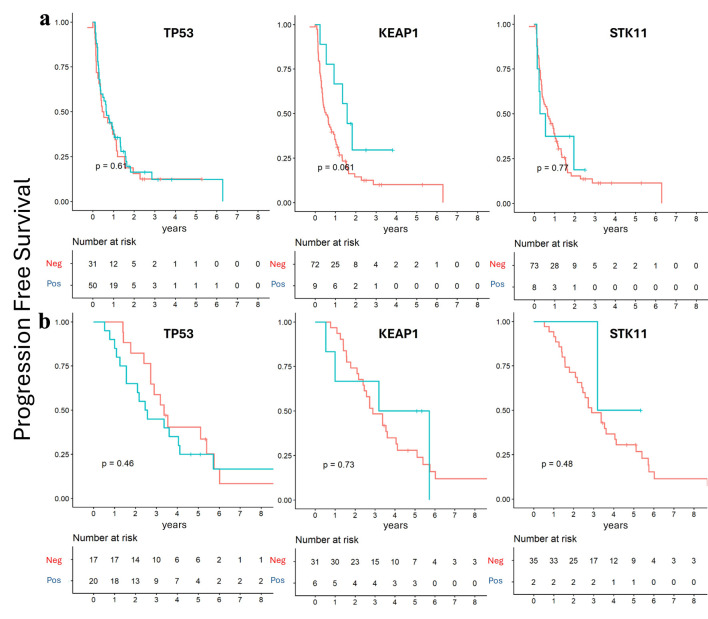
Progression-free survival according to mutation status. Kaplan–Meier survival analysis showed no significant survival differences according to TP53, STK11, and KEAP1 mutation status. (a) *De novo* group. (b) Recurrent group.

To further explore potential prognostic effects, patients were subsequently stratified by mutation status within each group. In the *de novo* group, TP53 and STK11 mutation statuses were not associated with significant differences in PFS and OS, while KEAP1 mutation showed a trend toward longer OS and PFS, approaching statistical significance (P = 0.06) (Figs. 4a and 5a). In contrast, within the recurrent group, TP53 mutation status was the only alteration associated with a trend toward poorer OS (P = 0.06) (Figs. 4b and 5b).

**Figure 6:**
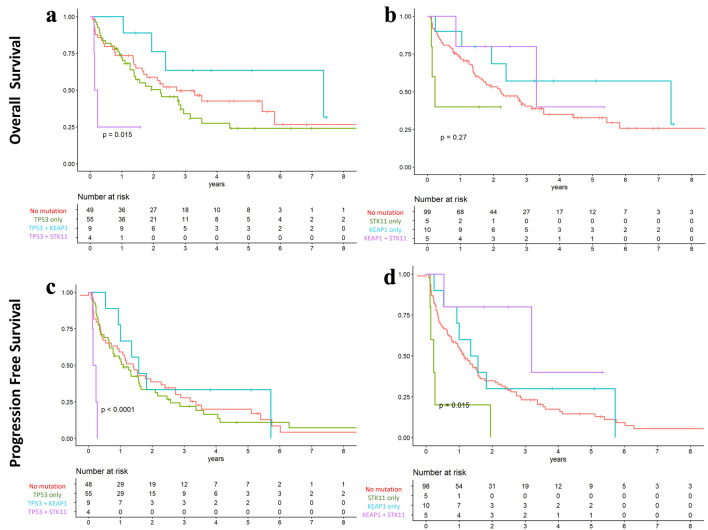
Overall and progression-free survival according to co-mutation status in the overall cohort. (a and c) TP53 with co-mutations in KEAP1 and STK11. (b and d) KEAP1 and STK11 co-mutations. Kaplan–Meier survival analysis demonstrated longer overall and progression-free survival in patients with TP53–KEAP1 and KEAP1–STK11 co-mutations compared with those harboring single-gene mutations.

The impact of co-mutations was analyzed across the entire cohort. For OS, KEAP1–STK11 co-mutation was not significantly associated with outcomes. However, TP53 + KEAP1 co-mutations were associated with longer median OS compared with TP53 mutations alone, while TP53 + STK11 co-mutations were associated with shorter OS (87.6 vs. 26.4 vs. 2.22 months, respectively; P = 0.015) (Fig. 6a).

For PFS, patients with TP53 + KEAP1 co-mutations had a longer median PFS compared with those with TP53 mutations alone, whereas TP53 + STK11 co-mutations were associated with shorter PFS (18.8 vs. 13.8 vs. 2.22 months, respectively; P < 0.001) (Fig. 6b). Similarly, those harboring KEAP1−STK11 co-mutation demonstrated longer median PFS compared to either mutation alone (38.2 vs. 13.8 months for KEAP1 and 2.76 months for STK11; P = 0.015) (Fig. 6d).

When analyses were stratified within each mutation group, TP53 + KEAP1 still showed significantly longer PFS compared to TP53 + STK11 and TP53 mutant tumors (18.8 vs. 2.22 vs. 7.6 months, respectively; P = 0.002) and trended towards longer OS compared to other subgroups (Supplementary Material 2, wjon.elmerpub.com). No significant associations were observed within the recurrent group (Supplementary Material 3, wjon.elmerpub.com).

## Discussion

This single-center real-world study evaluated the predictive and prognostic implications of TP53, STK11, and KEAP1 with clinical outcomes in actionable mutation–negative NSCLC patients. We observed no statistically significant differences in the incidence of these mutations between *de novo* and recurrent disease. However, near-significant associations were noted for KEAP1 mutations in *de novo* cases and TP53 mutations in recurrent disease, suggesting that the prognostic relevance of these alterations may reflect intrinsic tumor biology and potentially dynamic molecular evolution over the disease course.

Our findings are consistent with prior evidence demonstrating that the impact of these mutations on clinical outcomes is context-dependent. For example, a retrospective cohort study reported an association between TP53 mutations and shorter disease-free survival in patients with localized NSCLC, but not in those with advanced-stage disease [7]. Importantly, that study did not exclude patients with actionable oncogenic drivers. In contrast, a study focused on actionable mutation–negative NSCLC showed an association between TP53 mutations and inferior OS [13], highlighting the importance of genomic context in relation to prognostic significance.

The interaction between mutations is a known and increasingly recognizing phenomenon which has demonstrated to have significant biological and clinical considerations. As such, STK11 and KEAP1 mutations, which are frequently co-mutated, have been consistently associated with poor prognosis in specific clinical contexts and may have implications for therapeutic decision-making. Prior studies have demonstrated that co-mutation of STK11 and KEAP1 is associated with significantly shorter OS in patients receiving first-line systemic therapies [14, 15]. These findings have been replicated across diverse populations, including a Hispanic cohort, and have also been linked to differences in PD-L1 expression [16]. Interestingly, STK11-mutant tumors are typically characterized by low PD-L1 expression, whereas KEAP1-mutant tumors tend to exhibit higher PD-L1 levels; however, both converge on a profoundly immunosuppressive tumor microenvironment. This is particularly relevant, as immunotherapy-based regimens remain the cornerstone of management in actionable mutation–negative NSCLC [17, 18]. In this context, two phase III trials, POSEIDON and TRITON, enrolling predominantly actionable mutation–negative patients, are evaluating the benefit of dual immunotherapy combined with chemotherapy compared with single-agent immunotherapy plus chemotherapy [19]. Preliminary results from the POSEIDON trial demonstrated improved 5-year OS among patients with STK11 and KEAP1 mutations treated with durvalumab, tremelimumab, and chemotherapy, compared with durvalumab plus chemotherapy alone [19]. Notably, the specific impact of STK11/KEAP1 co-mutation has yet to be fully characterized in these studies. Collectively, these findings suggest that the presence of these mutations may represent an important consideration in the management of NSCLC, given their influence on tumor biology and treatment response [20]. In our cohort, STK11 and KEAP mutations were noticed to coexist in both *de novo* and recurrent groups, with KEAP1 mutations showing a trend toward longer OS and PFS in *de novo* patients. STK11-KEAP1 co-mutation was associated with longer PFS in the overall cohort, but was not associated with outcomes when stratified by disease status, supporting their role as prognostic rather than predictive biomarkers [11].

The relationships of TP53 with STLK11 and KEAP1 in lung cancer have also been described. Interestingly, one study reported that the co-occurrence of KEAP1 and TP53 mutations may be associated with better OS compared to KEAP1 mutation alone [7], while a study across multiple genomic datasets demonstrated that TP53-KEAP1–mutant lung adenocarcinoma tumors exhibit distinct immune microenvironment and pathway signatures, and are associated with improved survival compared with KEAP1-mutant tumors, approaching that observed in TP53-mutant tumors [21]. Similarly, TP53-STK11 co-mutations have been associated with higher tumor immune activity and were associated with improved response to immunotherapy [22]. However, these studies did not focus on actionable mutation–negative cases, which may influence these associations given the potential driver and genomic interactions. These findings underscore the complexity of mutational interactions. In our study, focusing exclusively on actionable mutation–negative NSCLC, TP53-KEAP1 co-mutation was associated with a longer OS and PFS compared to TP53, STK11A, or KEAP1 mutations alone. This effect, however, did not persist when analyses were stratified by disease status.

Several limitations should be acknowledged. First, the retrospective and single-center design may limit generalizability. Second, the sample size, particularly after exclusion of actionable mutation–positive cases, reduced our power to detect modest associations between subgroups and could lead to potential instability of estimates. Third, intrinsic differences between *de novo* and recurrent disease—including baseline disease status, prior treatment exposure, and underlying tumor biology—may independently influence survival and progression outcomes, making it challenging to isolate the specific effect of these mutations within this comparison. Fourth, the specific types of TP53 mutations were not collected; this is relevant as different mutations (in-frame, frameshift, or missense) have distinct biologic effects, which may have implications in this scenario. In addition, the distinction between *de novo* and recurrent disease can be challenging to define retrospectively, as classification may depend on prior imaging, treatment history, and available clinical documentation, potentially introducing misclassification bias. The variability of clinical contexts across the available literature portrays the challenges of incorporating these mutations into routine clinical decision-making, as their associations with progression, recurrence, and treatment response are inconsistent. Nevertheless, their recurrent associations with shorter overall and disease-free survival underscore the need to further characterize these mutations in larger prospective studies, which may lead to improved risk stratification and support more tailored or aggressive treatment approaches for patients with actionable mutation–negative NSCLC.

### Conclusion

In this single-center real-world study, TP53, STK11, and KEAP1 mutations occurred at similar frequencies in *de novo* and recurrent actionable mutation–negative NSCLC and were not observed to be associated with significant differences in clinical outcomes based on disease presentation. Collectively, these findings suggest the interpretation of TP53, STK11, and KEAP1 as context-dependent prognostic, rather than predictive, biomarkers, likely reflecting intrinsic tumor biology rather than disease status. Larger prospective studies are warranted to further elucidate the role of these mutations in actionable mutation–negative NSCLC.

## Supplementary Material

Suppl 1 Mutation analysis of actionable mutation–negative NSCLC cases.

Suppl 2 Overall and progression-free survival according to co-mutation status in the *de novo* cohort.

Suppl 3 Overall and progression-free survival according to co-mutation status in the recurrent group.

## Data Availability

The data supporting the findings of this study are available from the corresponding author upon reasonable request.

## Abbreviations

CI: confidence interval
ECOG: Eastern Cooperative Oncology Group
HR: hazard ratio
mOS: median overall survival
NGS: next-generation sequencing
NSCLC: non-small cell lung cancer
OR: odds ratio
OS: overall survival
PFS: progression-free survival

## References

[1] Siddique F, Shehata M, Ghazal M, Contractor S, El-Baz A (2024). Lung cancer subtyping: a short review. Cancers (Basel).

[2] Simarro J, Perez-Simo G, Mancheno N, Ansotegui E, Munoz-Nunez CF, Gomez-Codina J, Juan O (2023). Impact of molecular testing using next-generation sequencing in the clinical management of patients with non-small cell lung cancer in a public healthcare hospital. Cancers (Basel).

[3] Nindra U, Pal A, Bray V, Yip PY, Tognela A, Roberts TL, Becker TM (2024). Utility of multigene panel next-generation sequencing in routine clinical practice for identifying genomic alterations in newly diagnosed metastatic nonsmall cell lung cancer. Intern Med J.

[4] Ramalingam SS, Vansteenkiste J, Planchard D, Cho BC, Gray JE, Ohe Y, Zhou C (2020). Overall survival with osimertinib in untreated, EGFR-mutated advanced NSCLC. N Engl J Med.

[5] Kris MG, Johnson BE, Berry LD, Kwiatkowski DJ, Iafrate AJ, Wistuba, II, Varella-Garcia M (2014). Using multiplexed assays of oncogenic drivers in lung cancers to select targeted drugs. JAMA.

[6] Rocca A, Crino L, Braga L, Salton F, Ruaro B, Confalonieri M, Generali D (2025). Refining treatment strategies for non-small cell lung cancer lacking actionable mutations: insights from multi-omics studies. Br J Cancer.

[7] Saleh MM, Scheffler M, Merkelbach-Bruse S, Scheel AH, Ulmer B, Wolf J, Buettner R (2022). Comprehensive analysis of TP53 and KEAP1 mutations and their impact on survival in localized- and advanced-stage NSCLC. J Thorac Oncol.

[8] de Castro G, Kudaba I, Wu YL, Lopes G, Kowalski DM, Turna HZ, Caglevic C (2023). Five-year outcomes with pembrolizumab versus chemotherapy as first-line therapy in patients with non-small-cell lung cancer and programmed death ligand-1 tumor proportion score >/= 1% in the KEYNOTE-042 study. J Clin Oncol.

[9] Garassino MC, Gadgeel S, Speranza G, Felip E, Esteban E, Domine M, Hochmair MJ (2023). Pembrolizumab plus pemetrexed and platinum in nonsquamous non-small-cell lung cancer: 5-year outcomes from the phase 3 KEYNOTE-189 study. J Clin Oncol.

[10] Rustamov R, Fuzesi L, Lesser T, Tauscher D, Funke U, Elsner P, Mireskandari M (2026). Prevalence and clinico-morphological correlates of STK11 mutations in a large cohort of NSCLC lung adenocarcinomas. Virchows Arch.

[11] Swanton C, Govindan R (2016). Clinical implications of genomic discoveries in lung cancer. N Engl J Med.

[12] https://www.R-project.org/.

[13] Chi J (2024). Characterization of TP53 mutations in actionable driver-negative non-small cell lung cancer (NSCLC). J Clin Oncol.

[14] Julian C, Pal N, Gershon A, Evangelista M, Purkey H, Lambert P, Shi Z (2023). Overall survival in patients with advanced non-small cell lung cancer with KRAS G12C mutation with or without STK11 and/or KEAP1 mutations in a real-world setting. BMC Cancer.

[15] Papillon-Cavanagh S, Doshi P, Dobrin R, Szustakowski J, Walsh AM (2020). STK11 and KEAP1 mutations as prognostic biomarkers in an observational real-world lung adenocarcinoma cohort. ESMO Open.

[16] Cordeiro de Lima VC, Corassa M, Saldanha E, Freitas H, Arrieta O, Raez L, Samtani S (2022). STK11 and KEAP1 mutations in non-small cell lung cancer patients: Descriptive analysis and prognostic value among Hispanics (STRIKE registry-CLICaP). Lung Cancer.

[17] Xu K, Lu W, Yu A, Wu H, He J (2024). Effect of the STK11 mutation on therapeutic efficacy and prognosis in patients with non-small cell lung cancer: a comprehensive study based on meta-analyses and bioinformatics analyses. BMC Cancer.

[18] Skoulidis F, Araujo HA, Do MT, Qian Y, Sun X, Galan-Cobo A, Le JT (2024). CTLA4 blockade abrogates KEAP1/STK11-related resistance to PD-(L)1 inhibitors. Nature.

[19] Skoulidis F, Araujo HA, Do MT, Qian Y, Sun X, Cobo AG, Le JT (2025). Author correction: CTLA4 blockade abrogates KEAP1/STK11-related resistance to PD-(L)1 inhibitors. Nature.

[20] Shiller M, Johnson M, Auber R, Patel SP (2024). Clinical perspectives on the value of testing for STK11 and KEAP1 mutations in advanced NSCLC. Front Oncol.

[21] Scalera S, Mazzotta M, Corleone G, Sperati F, Terrenato I, Krasniqi E, Pizzuti L (2021). KEAP1 and TP53 frame genomic, evolutionary, and immunologic subtypes of lung adenocarcinoma with different sensitivity to immunotherapy. J Thorac Oncol.

[22] Naqash AR, Floudas CS, Aber E, Maoz A, Nassar AH, Adib E, Choucair K (2024). Influence of TP53 comutation on the tumor immune microenvironment and clinical outcomes with immune checkpoint inhibitors in STK11-mutant non-small-cell lung cancer. JCO Precis Oncol.

