# Supplementary material for: Incidence and Prognostic Value of TP53, STK11, and KEAP1 Mutations Between De Novo Versus Recurrent Actionable Mutation–Negative Non-Small Cell Lung Cancer: A Single-Center Retrospective Study
Source: World J Oncol. 2026 May 8;17(3):310–21. doi: 10.14740/wjon2761 (PMC13171259; doi:10.14740/wjon2761)
Supplement: Suppl 1 — Mutation analysis of actionable mutation–negative NSCLC cases. [file wjon-17-03-310-s001.docx]

**Suppl 1.** Mutation analysis of actionable mutation–negative NSCLC cases

| **Factors**  **(Ranked)** | | | | | | ***De Novo***  **(n = 82)** | | | | | | | | | | | | | **Recurrent**  **(n = 37)** | | | | | | | | | | | | | | | **P value** | | | | | | | | | |
| --- | --- | --- | --- | --- | --- | --- | --- | --- | --- | --- | --- | --- | --- | --- | --- | --- | --- | --- | --- | --- | --- | --- | --- | --- | --- | --- | --- | --- | --- | --- | --- | --- | --- | --- | --- | --- | --- | --- | --- | --- | --- | --- | --- |
| **TP53 status**  **Mutated**  **Wild-type** | | | | | | 50 (61%)  32 (39%) | | | | | | | | | | | | | | | | | | 20 (54%)  17 (46%) | | | | | | | | | | 0.54 | | | | | | | | | |
| **KEAP 1 status**  **Mutated**  **Wild-type** | | | | | | 9 (11%)  73 (89%) | | | | | | | | | | | | | | | | | | 6 (16%)  31 (84%) | | | | | | | | | | 0.55 | | | | | | | | | |
| **STK 11 status**  **Mutated**  **Wild-type** | | | | | | 8 (10%)  74 (90%) | | | | | | | | | | | | | | | | | 2 (5%)  35 (95%) | | | | | | | | | | 0.72 | | | | | | | | | |  |
| **PTEN** | | | | | |  | | | | | | | | | | | | | | | |  | | | | | | | | | |  | | | | | | | | | |  |  |
| **Mutated**  **Wild-type** | | | | | | 2 (2%)  80 (98%) | | | | | | | | | | | | | | | 4 (11%)  33 (89%) | | | | | | | | | | 0.07 | | | | | | | | | |  |  |  |
| **NFE2L2** | | | | | |  | | | | | | | | | | | | | | |  | | | | | | | | | |  | | | | | | | | | |  |  |  |
| **Mutated**  **Wild-type** | | | | | | 5 (6%)  77 (94%) | | | | | | | | | | | | | | 1 (3%)  36 (97%) | | | | | | | | | | 0.66 | | | | | | | | | |  |  |  |  |
| **PIK3CA** | | | | | |  | | | | | | | | | | | | | |  | | | | | | | | | |  | | | | | | | | | |  |  |  |  |
| **Mutated**  **Wild-type** | | | | | | | | | 4 (5%)  78 (95%) | | | | | 0  37 (100%) | | | | | | | | | | 0.3 | | | | | | | | | |  |  |  |  |  |  |  |  |  |  |
| **RB1** | | | | | | | | |  | | | | |  | | | | | | | | | |  | | | | | | | | | |  |  |  |  |  |  |  |  |  |  |
| **Mutated**  **Wild-type** | | | | | | | | | 3 (4%)  79 (96%) | | | | | 1 (3%)  36 (97%) | | | | | | | | | | 0.3 | | | | | | | | | |  |  |  |  |  |  |  |  |  |  |
| **KMT2C** | | | | | | | | |  | | | | |  | | | | | | | | | |  | | | | | | | | | |  |  |  |  |  |  |  |  |  |  |
| **Mutated**  **Wild-type** | | | | | | | | | 2 (2%)  80 (98%) | | | | | 1(3%)  36 (97%) | | | | | | | | | | 1 | | | | | | | | | |  |  |  |  |  |  |  |  |  |  |
| **SMARCA4** | | | | | | | | |  | | | | |  | | | | | | | | | |  | | | | | | | | | |  |  |  |  |  |  |  |  |  |  |
| **Mutated**  **Wild-type** | | | | | | | | | 2 (2%)  80 (98%) | | | | | 1 (3%)  36 (97%) | | | | | | | | | | 1 | | | | | | | | | |  |  |  |  |  |  |  |  |  |  |
| **FGFR2** | | | | | | | | |  | | | | |  | | | | | | | | | |  | | | | | | | | | |  |  |  |  |  |  |  |  |  |  |
| **Mutated**  **Wild-type** | | | | | | | | | 1 (1%)  81 (99%) | | | | | 1 (3%)  36 (97%) | | | | | | | | | | 0.53 | | | | | | | | | |  |  |  |  |  |  |  |  |  |  |
| **ATM** | | | | | | | | |  | | | | |  | | | | | | | | | |  | | | | | | | | | |  |  |  |  |  |  |  |  |  |  |
| **Mutated**  **Wild-type** | | | | | | | | | 1 (1%)  81 (99%) | | | | | | | | | | 0  37 (100%) | | | | | | | | | | | | | | | 1 | | | | | | | | | |
| **MEN** | | | | | | | | |  | | | | | | | | | |  | | | | | | | | | | | | | | |  | | | | | | | | | |
| **Mutated**  **Wild-type** | | | | | | | | | 1 (1%)  81 (99%) | | | | | | | | | | 0  37 (100%) | | | | | | | | | | | | | | | 1 | | | | | | | | | |
| **SMAD4** | | | | | | | | |  | | | | | | | | | |  | | | | | | | | | | | | | | |  | | | | | | | | | |
| **Mutated**  **Wild-type** | | | | | | | | 0  82 (100%) | | | | | | | | | | | 0  37 (100%) | | | | | | | | | | | | | | | 1 | | | | | | | | | |
| **NTUM1** | | | | | | | |  | | | | | | | | | | |  | | | | | | | | | | | | | | |  | | | | | | | | | |
| **Mutated**  **Wild-type** | | | | | | | 0  82 (100%) | | | | | | | | | | | | 0  37 (100%) | | | | | | | | | | | | | | | 1 | | | | | | | | | |
|  | | | | | | |  | | | | | | | | | | | |  | | | | | | | | | | | | | | |  | | | | | | | | | |
