# Supplementary figures and images for: Incidence and Prognostic Value of TP53, STK11, and KEAP1 Mutations Between De Novo Versus Recurrent Actionable Mutation–Negative Non-Small Cell Lung Cancer: A Single-Center Retrospective Study
Source: World J Oncol. 2026 May 8;17(3):310–21. doi: 10.14740/wjon2761 (PMC13171259; doi:10.14740/wjon2761)

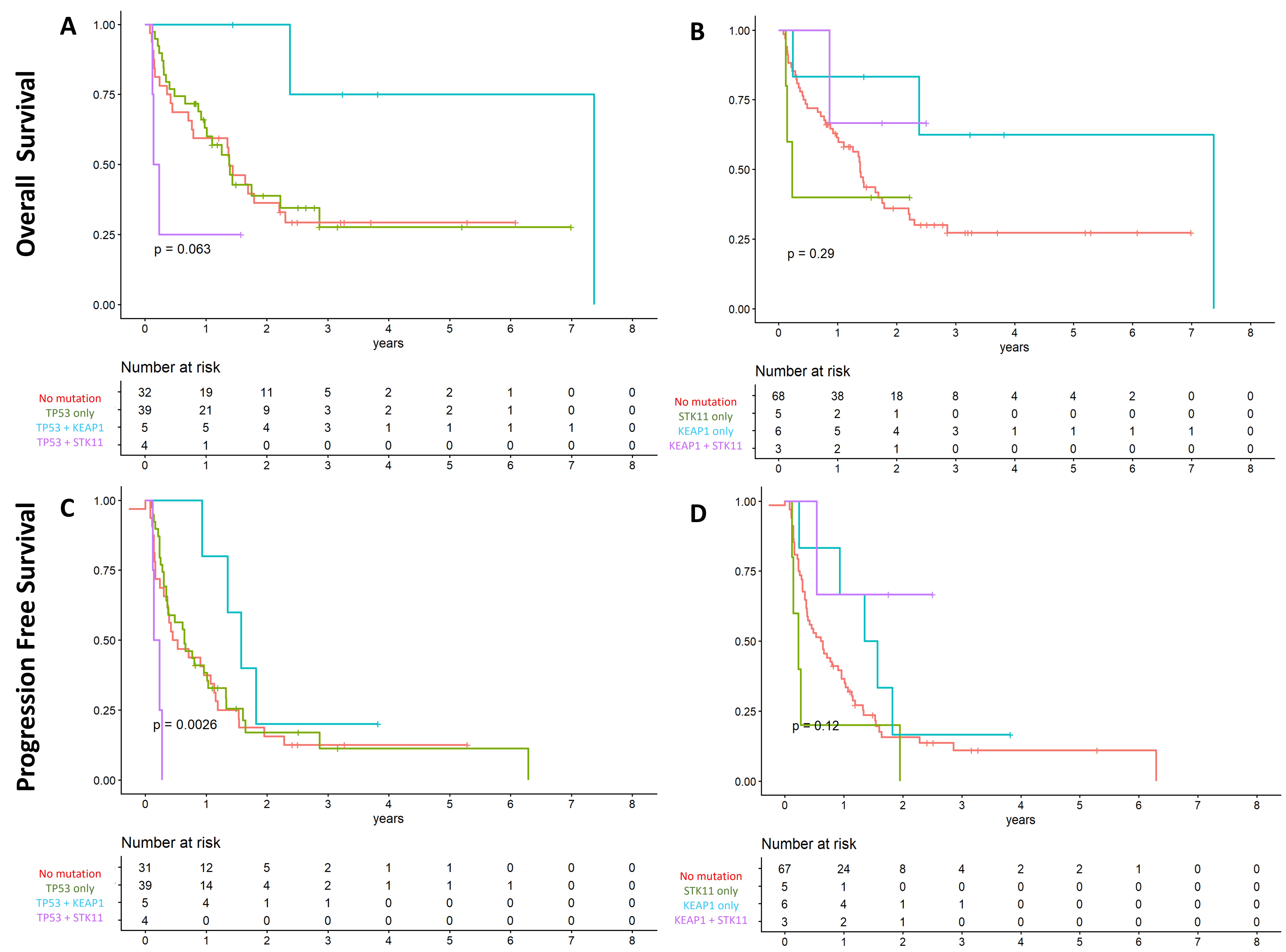

Supplement: Suppl 2 — Overall and progression-free survival according to co-mutation status in the de novo cohort. [file wjon-17-03-310-s002.jpg]

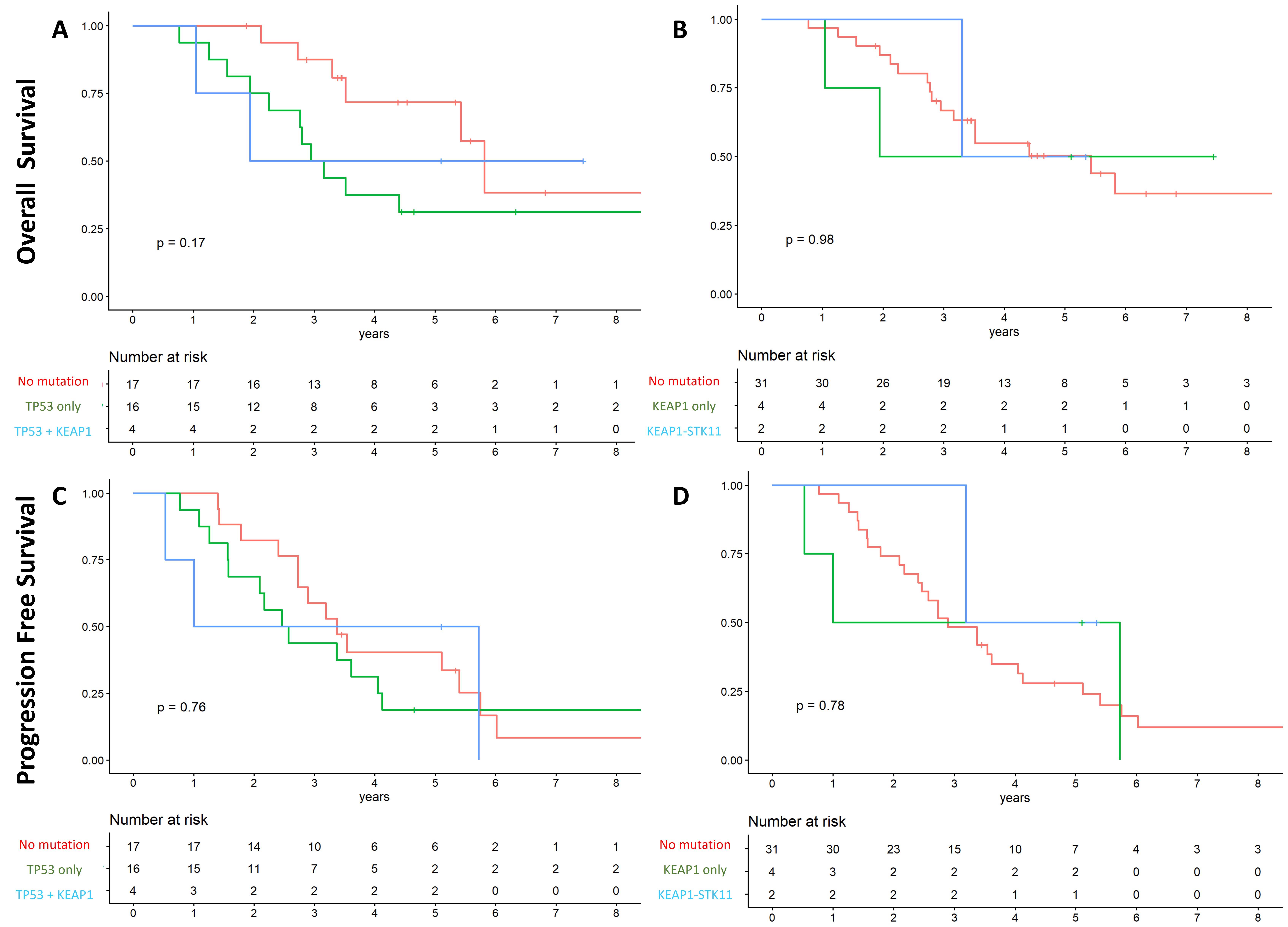

Supplement: Suppl 3 — Overall and progression-free survival according to co-mutation status in the recurrent group. [file wjon-17-03-310-s003.jpg]
